# Management of patients with severe asthma: results from a survey among allergists and clinical immunologists of the Central Italy Inter-Regional Section of SIAAIC

**DOI:** 10.1186/s12948-021-00160-x

**Published:** 2021-12-05

**Authors:** G. Carli, A. Farsi, S. Bormioli, E. Ridolo, F. Fassio, S. Pucci, M. Montevecchi, M. Riparbelli, L. Cosmi, P. Parronchi, O. Rossi

**Affiliations:** 1 SOS Allergologia ed Immunologia, USL Toscana Centro, Prato, Italy; 2 Dept. Experimental and Clinical Medicine, University of Florence, Florence, Italy; 3 SOD Immunoallergologia, AOU Careggi, Florence, Italy; 4 Dept. Medicine and Surgery, University of Parma, Parma, Italy; 5 SOC Allergologia ed Immunologia Clinica, Ospedale San Giovanni di Dio, USL Toscana Centro, Florence, Italy; 6 OUC Allergologia, PO Civitanova Marche, Civitanova Marche, MC Italy; 7 GSK Medical Department, Verona, Italy; 8 SOD Immunologia e Terapie cellulari, AOU Careggi, Florence, Italy

**Keywords:** Asthma, Allergy, Adherence, Biologic therapy, ICS/LABA, Corticosteroids, Allergists

## Abstract

**Background:**

Asthma, and severe asthma in particular, is often managed within a specialized field with allergists and clinical immunologists playing a leading role. In this respect, the National Scientific Society SIAAIC (*Società Italiana di Allergologia, Asma ed Immunologia Clinica*), structured in Regional and Inter-Regional sections, interviewed a large number of specialists involved in the management of this respiratory disease.

**Methods:**

A survey entitled “Management of patients with asthma and severe asthma” based on 17 questions was conducted through the SIAAIC newsletter in 2019 thanks to the collaboration between GlaxoSmithKline S.p.A. and the Inter-Regional Section of SIAAIC of Central Italy.

**Results:**

Fifty-nine allergists and clinical immunologists participated to the survey, and 40 of them completed the entire questionnaire. Almost all of the specialists (88%) reported that asthma control was achieved in above 50% of their patients, even if only one third (32%) actually used validated clinical tools such as asthma control test (ACT). Poor adherence to inhaled therapy was recognized as the main cause of asthma control failure by 60% of respondents, and 2–5 min on average is dedicated to the patient inhaler technique training by two-thirds of the experts (65%). Maintenance and as-needed therapy (SMART/MART) is considered an appropriate approach in only a minority of the patients (25%) by one half of the respondents (52%). A high number of exacerbations despite the maximum inhalation therapy were recognized as highly suspicious of severe asthma. Patients eligible for biological therapies are 3–5% of the patients, and almost all the responders (95%) agreed that patients affected by severe asthma need to be managed in specialized centers with dedicated settings. Biological drugs are generally prescribed after 3–6 months from the initial access to the center, and once started, the follow-up is initially programmed monthly, and then every 3–6 months after the first year of treatment (96% of responders). After phenotyping and severity assessment, comorbidities (urticaria, chronic rhinosinusitis with or without nasal polyps, vasculitis, etc.) are the drivers of choice among the different biological drugs. In the management of severe asthma, general practitioners (GPs) should play a central role in selecting patients and referring them to specialized centers while Scientific Societies should train GPs to appropriately recognize difficult asthma and promote public disease awareness campaigns.

**Conclusions:**

This survey which collects the point of view of allergists and clinical immunologists from Central Italy highlights that asthma control is still not measured with validated instruments. There is a general consensus that severe asthma should be managed only in dedicated centers and to this aim it is essential to encourage patient selection from a primary care setting and develop disease awareness campaigns for patients.

## Introduction

Asthma is a common chronic inflammatory disease of the lower airways affecting between 5 and 10% of the general population in the European countries. Most asthmatics can be treated with low- or medium-dose inhaled corticosteroids (ICS), but approximately 10% of patients suffer from a more severe form of the disease, thus requiring stronger treatments such as high-dose ICS combined with long-acting beta-2 agonist (LABA), oral anti-leukotrienes and long-acting muscarinic antagonists (LAMA). Refractory asthma regardless of maximal aimed inhaled therapy, requires the use of systemic steroids and biological drugs [1].

While both specialized centers and general practitioners (GPs) are engaged in low/moderate asthma cases, severe asthma is usually managed by pneumologists, allergists and clinical immunologists in highly specialized centers. Unexpectedly, and often due to a lack of patients’ awareness or perception of their disease, even mild and moderate forms of asthma are not sufficiently controlled often due to inadequate adherence to treatment. As a consequence, increase in exacerbations, a greater need of health resources (i.e. urgent visits and/or hospitalizations), higher social costs and, in extreme cases, even fatal events can be observed.

The Inter-Regional Section of SIAAIC (Società Italiana di Allergologia, Asma ed Immunologia Clinica) which includes Tuscany, Emilia-Romagna, Republic of San Marino, Umbria and Marche, carried out a survey amongst its members to screen how immunologists/allergists manage the disease at all severity levels and how they perceive the level of asthma control. The final aim was to assess knowledge, attitudes and practices of the participants in order to identify possible grey areas in the management of this disease and new ideas to design training projects aimed to improve asthma management.

## Materials and methods

In 2019, Inter-Regional Section of Tuscany, Emilia-Romagna, Republic of San Marino, Umbria and Marche of SIAAIC carried out a survey entitled “Management of patients with asthma and severe asthma” to investigate knowledge, opinions, and practices of immunologists and allergy specialists about asthma and severe asthma.

A group of SIAAIC experts produced 17 questions which were submitted to the members of the Inter-Regional Section between the 20th of May and the 15th of September 2019.

Questions were uploaded to an electronic platform developed by GSK’s Knowledge Center at WNS Global Services (P) Limited, based in India. Participation in the survey did not involve the collection of any personal data from the participants. The results were collected and processed by Knowledge Center and reported in an aggregated form to GSK, who sent them back to the Scientific Society without any possibility of tracing identity and origin of the participants or matching the provided answers to any subject.

The intellectual property of the results of the survey is an exclusive right of the Inter-Regional Section of SIAAIC.

## Results

Fifty-nine allergists and clinical immunologists of the Inter-Regional Section of SIAAIC from Tuscany, Emilia-Romagna, Republic of San Marino, Umbria and Marche participated to the survey. Forty completed the entire questionnaire. The geographic area of this group of specialists covers about 1,055,000 inhabitants, on a total Italian population of about 60,300,000.

Complete results are described in Table 1.

**Table 1:** Results of the survey “Management of patients with asthma and severe asthma” of the Inter-Regional Section of SIAAIC

Questions	% (N° resp)
1. Which is the percentage of asthmatics you are following and treating who are controlled?	
• Less than 30%	2% (1)
• 30 to 50%	10% (4)
• More than 50%	88% (35)
2. Which is the tool you use to evaluate asthma control in outpatient patients?	
• ACT	32% (13)
• PEF monitoring	0% (0)
• Interview	35% (14)
• Spirometry	32% (13)
• FeNO	0% (0)
3. Which is the most frequent cause of uncontrolled asthma in the real-life?	
• Inadequate management of comorbidities (gastroesophageal reflux, nasal polyposis, obesity, …)	15% (6)
• Non-adherence to treatment	60% (24)
• Incorrect use of devices	8% (3)
• Inadequate therapy in relation to severity level	5% (2)
• Lack of background therapy	12% (5)
4. Which is the best method in your opinion to monitor adherence to background therapy (ICS, LABA, LAMA, anti-leukotriene)?	
• Ask the patient directly	62% (25)
• Ask the general practitioner to verify numbers of drug prescriptions through the database	12% (5)
• Use of Smart devices able to monitor drug usage	15% (6)
• Adherence cannot be monitored	0% (0)
• By FeNO measurement	10% (4)
5. How long do you spend time (on average) to explain the correct use of the inhalation device?	
• Between 2 and 5 min	65% (26)
• Over 5 min	22% (9)
• I don't always have time to explain the device	12% (5)
• Demand to the general practitioner	0% (0)
6. In your clinical experience, how many patients may be treated with flexible doses of inhalation therapy, according to the MART/SMART scheme?	
• 0–25%	52% (21)
• 25–50%	25% (10)
• 50–75%	20% (8)
• 75–100%	2% (1)
7. In your clinical practice which one of the following definitions is the first to identify a patient with severe asthma?	
• Uncontrolled patient with medium–high doses ICS + other controller	15% (6)
• Patient continuously treated with oral steroids for at least 6 months	2% (1)
• Frequent exacerbating patient despite maximal treatment	55% (22)
• Patient with frequent access to Emergency Department and/or hospitalization	5% (2)
• Patient treated with medium–high doses of ICS-LABA and frequent use of drug as needed (3–4 puffs/day)	22% (9)
8. In your clinical experience, how many asthmatic patients can be suitable for current biological therapies?	
• Less than 3%	35% (14)
• Ranging between 3 and 5%	58% (23)
• Above 5%	8% (3)
9. In your opinion, how should patients with severe refractory asthma be managed?	
• During normal outpatient activity	5% (2)
• Organizing dedicated severe asthma clinics in the same hospital	60% (24)
• It would be better to send patients with severe asthma to organized centers with a high specialized background	35% (14)
10. How long after following a patient with severe asthma refractory to maximal standard therapy (ICS, LABA, LAMA, leukotriene) do you evaluate the option of biologic therapy?	
• Since the first visit, if the patient shows the inclusion criteria for a biologic drug	42% (17)
• I follow the patient changing therapies for 3–6 months and then I evaluate the biological treatment	52% (21)
• I wait up to a year before considering biologic treatment	5% (2)
11. As a specialist, which one of the following is the main therapeutic goal of a biologic therapy for severe asthma?	
• Reduction in exacerbations	32% (13)
• Reduction of systemic steroid intake	18% (7)
• Improvement of quality of life	25% (10)
• Improvement of asthma control, assessed through ACT	12% (5)
• Improvement of respiratory function	12% (5)
12. Which is the main result that patient with severe refractory asthma expects from inhalation treatment?	
• Reduced number of exacerbations	15% (6)
• Reduced need of emergency department visits or hospitalization for asthma	8% (3)
• Reduced use of systemic steroids with therefore reduced side effects	2% (1)
• Reduced symptoms limiting the everyday quality of life (e.g. sleep quality, effort dyspnoea,…)	75% (30)
13. Once you have started a biologic therapy how often do you follow the patient during the first year?	
• The patient is visited every month with personal verification of both the course of therapy and clinical outcomes	45% (18)
• The patient is fully re-evaluated once a month in the first 3–6 months, then every 6 months	52% (21)
• Once therapy is set up, visits are arranged every 6 months. In the meantime, the patient’s assessment is carried out by the nursing staff	2% (1)
14. In your opinion, once stabilization is achieved in a severe asthmatic patient, how often is the outpatient monitoring necessary?	
• Every 3 months	48% (19)
• Every 6 months	48% (19)
• Every 12 months	5% (2)
15. To date in Italy anti IgE and anti IL5 biologics are available in well-codified phenotypes of asthma. Which is the basis of your choice in the case of patients with inclusion criteria for both therapies?	
• Whenever both biologics are indicated, I prefer anti IgE because I think that allergy is the main driver of the inflammatory process	15% (6)
• Whenever both biologics are indicated I prefer anti IL5 because I consider eosinophils the main drivers of the inflammatory process	10% (4)
• I choose accordingly to the patient's comorbidities (e.g. presence of eosinophilic nasal polyposis, vasculitis, urticaria, etc.)	72% (29)
• I choose accordingly to practical aspects: cost NHS therapy, posology, etc.	2% (1)
16. Which is the role of the general practitioner in the management of patients with severe asthma?	
• Encourage the early recognition of these patients to be sent to specialized centers	28% (11)
• Monitor adherence to the background therapy	2% (1)
• Monitor patient's clinical status, e.g. systematically collecting agreed parameters	5% (2)
• All previous	65% (26)
17. In your opinion, which one could be the first feasible action promoted by a Scientific Society to favour awareness of the patients about their pathology and possible therapeutic choices?	
• To carry on meetings with patient groups or patient associations	20% (8)
• To produce training booklets to be delivered to patient or care-giver at the time of the visit	8% (3)
• To promote training courses for general practitioners	48% (19)
• To carry on media campaigns of disease awareness	25% (10)

## Discussion

The survey about the management of asthma and severe asthma has been carried out on a representative sample of allergists/immunologists located in the central Regions of Italy. Its results highlight that management of patients with asthma by specialists correlates with a better control of the disease (above 50% according to 88% of the respondents). Assessment of asthma control is achieved addressing questions to the patient by one third of the specialists, whereas a similar percentage of them collect structured information using a validated clinical tool (ACT). Finally, a further substantial part of the respondents uses the measurement of FEV1, even though this item is not truly recommended for this purpose by steering documents and it should be used to find the so called ‘personal best’ of the patient and to assess risk evaluation [1]. As a fact, current validated tools for the assessment of asthma control may not be adequate for all the patients with personalized interview possibly resulting as more informative (i.e. in differential diagnosis of other causes of dyspnea) whereas FEV1 might be better in the poor perceivers [2].

Irrespective of the measures used, lack of adherence to the inhalation therapy is recognized as the most frequent reason of uncontrolled asthma by the majority of the specialists [3]. As a matter of fact, adherence is low in chronic respiratory diseases such as COPD, not exceeding 19.9% and lower in comparison with other chronic diseases [4]. Comorbidities such as rhinitis, rhinosinusitis, gastroesophageal reflux, obesity, obstructive sleep apnea syndrome, depression and anxiety, if not properly treated, are additional key factors against asthma control [1]. Rhinitis may affect both allergic and non-allergic asthmatics, whereas the prevalence in the general population of chronic sinusitis and chronic rhinosinusitis with polyposis was 12% and 4%, respectively [5, 6].

The proper use of devices is a crucial factor for a successful therapy. Poor or improper inhaler technique in asthma is known to be associated with lack of control, and increased hospital visits [7, 8]. Moreover, using more than a single kind of inhaler increases the numbers of mistakes [8]. This element should be carefully considered when choosing the device, and an appropriate training time is recommended. Most of the respondents of the survey spend between 2 and 5 min to instruct their patients, whereas a low percentage (12%) do not because of lack of time. Time to teach is crucial, as the percentage of patients correctly using inhalers sharply increases from 24 to 79% if training time lasts 6 min [9, 10]. Following the GINA document [1], from STEP 3 on ICS/Formoterol as maintenance plus as needed (SMART/MART) can represent an alternative regimen to ICS-LABA as maintenance plus SABA as needed. Flexible treatment according to the SMART/MART approach is considered as helpful in 25% of the patients by one half of the specialists. In uncontrolled asthma, improvement in symptoms and quality of life rather than prevention of exacerbations or OCS use as consequences are perceived as the main expectations of patients. This might also affect their choice to shift from maintenance to symptomatic treatment.

Asthma is considered as severe when high numbers of exacerbations occur despite the maximal inhalation therapy by one half of the participants (55%) with minimal consideration of the OCS use (2% of responders) or hospitalization (5%). According to the Severe Asthma Network Italy Register (SANI), about two thirds of the Italian severe asthmatics (64%) are treated with OCS [11]. Further, the daily dose is higher than 10 mg prednisolone on average [11] despite the fact that ≤ 2.5 mg per day has been recently recommended [12]. The reduction of OCS is considered a benefit of biological treatment by a minority of the responders, while exacerbation reduction and quality of life improvements are favored targets of treatment. Actually, GINA document recommends OCS use within STEP 5 due to its potential side effects [13].

The percentage of patients with severe refractory asthma eligible for biological therapies is largely considered below 5% not different from the international value (approximately 3.7%) [13]. When undergoing biological treatment, there was a consensus to implement strict follow-up regimens in specialized centers and a following regular check in specialized setting is recommended by GINA [13]. At the time the survey was administered, only anti-IgE and anti-IL5/IL-5R strategies were available, with possible overlapping inclusion criteria [14]. It is of note that the majority of allergists and immunologists based their choice on an overall assessment of the patient starting from phenotyping and degree of disease severity, but which also includes comorbidities, in a comprehensive vision of asthma.

From the point of view of a Scientific Society like SIAAIC, two main areas of intervention were envisaged to improve asthma management. First, to improve patients awareness of their disease with joint campaigns with patients’ Associations and other Scientific Organizations. Secondly, to promote early specialist referring of patients with training events for general Practitioners aimed to update asthma management, enable early identification of difficult patients and optimize quality of care frameworks. In this way general Practitioners would be key players for the management of asthmatic patients, as detecting changes in the clinical course of the disease, prompting adherence to prescribed treatments and quickly referring patients to specialized centers, when suspecting severe asthma.

This survey represents the allergists/immunologists’ standpoint on the management of asthma and severe asthma. It would be interesting to compare these results with similar surveys gathering the opinion of other specialists, like pulmonologists or general practitioners.

## Data Availability

GSK’s Knowledge Center at WNS Global Services (P) Limited (India).
